# Seasonal variability of diet and trophic level of the gelatinous predator *Pelagia noctiluca* (Scyphozoa)

**DOI:** 10.1038/s41598-018-30474-x

**Published:** 2018-08-14

**Authors:** Giacomo Milisenda, Sergio Rossi, Salvatrice Vizzini, Veronica L. Fuentes, Jennifer E. Purcell, Uxue Tilves, Stefano Piraino

**Affiliations:** 1 Institute for Coastal Marine Environment (IAMC), National Research Council (CNR), Via L. Vaccara n 61, Mazara del Vallo (TP), 91026 Italy; 2 CONISMA - Consorzio Nazionale Interuniversitario per le Scienze del Mare, 00196 Rome, Italy; 3 Department of Biological and Environmental Sciences and Technologies, University of Salento, 73100 Lecce, Italy; 4 Department of Earth and Marine Sciences, University of Palermo, via Archirafi 18, 90100 Palermo, Italy; 5 Institut de Ciències del Mar, ICM-CSIC, PG. Maritim de la Barceloneta, 08003 Barcelona, Spain; 6 Western Washington University, Department of Biology, Bellingham, WA 98225 USA

## Abstract

Jellyfish populations apparently have increased in some places around the world and human problems with them also have increased. However, effects of jellyfish outbreaks in the ecosystems remain poorly understood and little or no information is available on their dietary preferences - in relation to the seasonal shifts of prey abundance - and on the potential variability of their impact on marine food webs. The mauve stinger *Pelagia noctiluca* (Forsskål, 1775) is by far the most common outbreak-forming scyphozoan jellyfish in the Western Mediterranean. By use of a combination of stomach contents, stable isotope (SI) and fatty acid (FA) analyses, we tested the hypothesis that changes in the seasonal dietary sources of *P*. *noctiluca* parallel changes in the FA and SI composition. Stomach content and biomarker analyses suggested that *P*. *noctiluca* is not a selective predator, cyclically shifting between carnivory and omnivory depending on the seasonality of accessible prey. The combination of SI and FA analyses highlighted the importance of microzooplankton as prey. Specific FA biomarkers showed that the diet of *P*. *noctiluca* changed seasonally depending on the availability of living plankton or suspended detritus. This study also revealed significant biochemical differences between jellyfish somatic and gonadal tissues, with total fatty acid concentration in the gonads up to ten times higher than in the somatic tissues.

## Introduction

Studies of trophic relationships help to unravel mechanisms of marine ecosystem functioning by illuminating foraging patterns of key species and related flows of energy and matter. Due to the inherent spatial and temporal variability of pelagic communities, tracing trophic relationships may be not as straightforward as in benthic habitats, leaving large gaps in the mechanistic interpretation of unpredicted biological events such as massive and recurrent outbreaks of gelatinous zooplankton communities in several coastal waters worldwide^1^. For instance, carnivorous jellyfish are mainly subject to bottom-up controls from their forage base rather than to top-down controls from predators^2^. This suggests that knowing what, how much, and where they eat would be key information to establish ecophysiological optima and trophic links supporting regime shifts in marine ecosystems coupled to local or temporary dominance of jellyfish. This knowledge is of particular importance for predicting the potential impact of jellyfish on marine ecosystem functioning and services, and eventually on human activities in coastal areas^3,4^.

A range of methods has been used to study trophic relationships in aquatic organisms, each with its own strengths and drawbacks. Stomach content analysis is the most direct approach^5,6^. This methodology may be useful to describe the diet and provide quantitative estimates of ingested prey. However, digestibility of prey differs significantly and identifiable items in stomach contents may often represent the most recently ingested food or prey requiring long digestion times^7^. Microzooplankton do not leave easily detectable remains and are therefore usually neglected, with the loss of key information^7,8^. An alternative approach to stomach content analysis could be experimental feeding incubations, which may give insights on food selectivity and ingestion rates, but suffer from artifact and over-simplification of complex natural food assemblages in the laboratory^9^. Indeed, the feeding rate of gelatinous species has often been underestimated in grazing experiments performed in artificial containers^6^. The use of identifiable molecular biomarkers, which pass from food sources to the consumer, is a complementary approach to detect small, soft-bodied prey, like ciliates and flagellates^10^. Such tracers may convey and integrate information on a consumer’s diet over days to months^10–12^, but separation of food sources is not straightforward. Each method is sensitive to different timescales and aspects of the diet, often leading to conflicting results on trophic relationships^13^. These biomarkers are complementary to the stomach content approach, but not sufficient in some cases when complex trophic interactions have to be described^7^.

Overall fatty acid composition and specific fatty acids (FAs) used as biomarkers can help to elucidate trophic relationships in food webs. Indeed, some FAs can only be synthesized *de novo* by certain taxa, especially by algae and bacteria, and then are conservatively transferred to the next trophic level unchanged or modified in an expected, detectable way^7,10,14^. For example, calanoid copepods are thought to be the only source of C20:1 (n-11 and n-9) and C22:1 (n-11 and n-9) monounsaturated fatty acids^15^; the identification of these FAs in the tissues of jellyfish suggests direct predation on copepods or other crustacean zooplankton^16^. However, knowledge of the FA metabolic pathways in many invertebrate taxa is in its infancy. FAs may be used successfully as qualitative trophic tracers in marine food webs to verify or identify predator–prey relationships, if the assumption that tracking FAs from source to consumers can be reliably met. To understand how food source contributions may vary seasonally or through the life span, stable isotope (SI) analysis becomes a powerful tool in the overall approach^17^. In fact, the complexity of food webs and the rarity of unique biomarkers impose uncertainties in the interpretation of FA data from field studies that must be resolved with complementary information such as stomach contents or stable isotopic data^7^.

The proportions of SIs of carbon (δ^13^C) and nitrogen (δ^15^N) may vary depending on the nutrient source, the physiology of the primary producers, and the trophic level of the consumers. SI analysis has successfully been used to elucidate food source partitioning^18,19^ and food web dynamics^20–22^. Its application in gelatinous zooplankton trophic ecology, however, is still infrequent^23–27^, but may help to identify trophic guilds and their potential prey sources in combination with various complementary methodologies^17^. So far, studies on the foraging ecology of the scyphozoan *Pelagia noctiluca* (Forsskål, 1775), the predominant gelatinous predator in the central and western Mediterranean Sea^28^, have relied solely on the analysis of stomach contents^27,29,30^, with a single exception^31^.

*Pelagia noctiluca* was first recorded in 1785 in the Strait of Messina^32^, at the confluence of the Southern Tyrrhenian and Ionian seas, where it now regularly undergoes massive population outbreaks with important effects on the structure and organization of the overall planktonic community^29^. Comparison of stomach contents and zooplankton composition indicate that this jellyfish is a generalist predator, with a clear seasonal variation in its diet^27,29^. The seasonal changes in diet may lead to seasonal changes in the composition of FAs and, as for *Aurelia aurita* in the North Sea, to the temporal variability in jellyfish δ^15^N and δ^13^C SI signatures^33^. Other factors like physiology, changes in behavior, investment in reproductive output also could affect FAs and SIs^34^. Thus, study of the seasonal variation of biochemical markers could help us to better understand their periodic reproduction events, because the biochemical composition of specific tissues, such as the gonads, may affect the quality of the breeding stock^35^. The simultaneous application of the three methodologies (e.g., stomach contents, FA and SI analyses) to clarify the trophic position of *P*. *noctiluca* and its trophic linkages (by predation or competition) with other taxa has not been attempted previously.

In this study, our objective was to clarify the predatory trophic impact of *P*. *noctiluca* medusae on the coastal marine food web by elucidating potential jellyfish dietary preferences over the year in relation to the seasonal shifts of prey availabilities. We used a combination of stomach contents, SI and FA analyses to test the hypothesis that changes in the seasonal dietary sources of *P*. *noctiluca* are paralleled by changes in the FA and SI composition of somatic and gonadic tissues, potentially accounting for the seasonal variations of reproductive and outbreak-forming potential of jellyfish.

## Results

### Stomach contents

The abundances of zooplankton taxa in the stomach contents of *P*. *noctiluca* and in the surface water layers were comparable (Table 1, Supplementary Information 1). Copepods were the most abundant group all year, followed by fish eggs and pteropods (Table 1).

**Table 1:** Monthly prey composition of zooplankton taxa in the stomach contents of Pelagia noctiluca.

		Nov (23)	Dec (17)	Feb (3)	Apr (12)	May (20)	Jun (27)	Jul (13)	Sep (13)
**Prey**	Siphonophores	0.04 ± 0.04	0 ± 0	0 ± 0	0 ± 0	0 ± 0	0 ± 0	0 ± 0	0.08 ± 0.0
	Pteropods	**0.70 ± 0.21+**	7.71 ± 3.65	0 ± 0	0.22 ± 0.22	0.45 ± 0.35	0.26 ± 0.11	0 ± 0	0 ± 0
	Ostracods	0.04 ± 0.04	**0 ± 0**	0 ± 0	0.11 ± 0.11	0.1 ± 0.06	0 ± 0	0.08 ± 0.07	0 ± 0
	Furcilia	0 ± 0	0 ± 0	0 ± 0	**0 ± 0 −**	0.05 ± 0.05	0 ± 0	0 ± 0	0 ± 0
	Fish larvae	0.04 ± 0.04	0.06 ± 0.05	0 ± 0	0 ± 0	0 ± 0	0.04 ± 0.03	0.08 ± 0.07	0 ± 0
	Fish eggs	**0.52 ± 0.16+**	**0.41 ± 0.12+**	0 ± 0	1.33 ± 0.57	**0.80 ± 0.22+**	**0.52 ± 0.16+**	0.08 ± 0.07	0 ± 0
	Copepods	**12.34 ± 3.45+**	6.65 ± 2.62	0.33 ± 0.33	36.66 ± 8.17	**21.95 ± 5.79+**	**10.37 ± 1.99+**	15.23 ± 9.95	1.38 ± 0.46
	Chaetognaths	0.43 ± 0.19	**0.60 ± 0.32+**	0 ± 0	0 ± 0	1.35 ± 0.70	0.22 ± 0.12	0 ± 0	0 ± 0
	Appendicularians	0.04 ± 0.04	0 ± 0	0 ± 0	0.11 ± 0.11	0.20 ± 0.15	0.07 ± 0.05	0 ± 0	0 ± 0

The numbers of jellyfish analyzed are in brackets beside each month. Values are expressed as mean number of prey medusa^−1^ ± standard error. Bold highlighted values indicate significant prey selection: “−” Significant negative selection; “+”positive significant selection. Significances were calculated using Chi-square test.

The total abundance of zooplankton prey in the stomach contents of *P*. *noctiluca* differed significantly over the year (Table 2). The highest values were in April and May, with 39 ± 8 (mean ± SE) and 25 ± 1 prey medusa^−1^, respectively. Copepods were consumed throughout the period of investigation, mostly in spring (April and May, 37 ± 8 and 22 ± 6 copepods medusa^−1^) and early summer (June and July, 10 ± 2 and 15 ± 9 copepods medusa^−1^), whereas few were found at the end of summer (September, 1 ± 0.5 copepods medusa^−1^) or in winter (February, 0.3 ± 0.3 copepods medusa^−1^) (Table 1). Pteropods were particularly abundant prey in December and May, while copepods and fish eggs were numerous in spring.

**Table 2:** One-way and 3-way PERMANOVA comparing (A) mean total prey (Total, prey medusa^−1^) and prey composition from stomach content analyses of *Pelagia noctiluca*; (B) mean δ^15^N and δ^13^C values from stable isotope analysis, and (C) total amount of fatty acids (ANOVA: Total), type of fatty acids (PERMANOVA: Saturated Fatty Acids, Monounsaturated Fatty Acids, and Polyunsaturated Fatty Acids: SFA-MUFA-PUFA), fatty acid composition for jellyfish and for plankton (FA composition).

	df	MS	F	p
**A**. **Stomach content**				
**Total**				
Month	8	1432	3.26	**
Residuals	128	438		
**Prey composition**				
Month	9	12.95	7.33	***
Residuals	139	1.76		
**B. Stable isotopes**				
**δ15N**				
Month	8	1.22	12.21	***
Residuals	36	0.11		
**δ13C**				
Month	8	2.92	27.93	***
Residuals	36	0.1		
**C**. **Fatty acids**				
**Total**				
Month	8	22.23	0.83	0.51
Sex	1	32.86	1.23	0.25
Bp	1	714	26.79	***
Month*Sex	8	7.14	0.26	0.94
Month*Bp	8	26.19	0.98	0.41
Sex*Bp	1	3.57	0.13	0.73
Month*Sex*Bp	6	7.58	0.28	0.9
Residuals	50	26.65		
**SFA-MUFA-PUFA**				
Month	8	714	0.93	0.47
Sex	1	1262	1.64	0.2
Bp	1	110	0.14	0.76
Month*Sex	8	1343	1.75	0.09
Month*Bp	8	1608	2.1	*
Sex*Bp	1	6153	8.04	**
Month*Sex*Bp	6	376	0.49	0.83
Residuals	50	764		
**FA composition (medusae)**				
Month	8	1308	1.65	*
Sex	1	2251	2.83	*
Bp	1	6793	8.56	***
Month*Sex	8	608	0.76	0.76
Month*Bp	8	1164	1.46	0.08
Sex*Bp	1	5476	6.9	***
Month*Sex*Bp	6	606	0.76	0.74
Residuals	50	792		
**FA composition (plankton)**				
Season	2	2487	3.16	**
Plankton Size	2	1143	1.45	0.19
Season*Plankton. Size	4	1037	1.32	0.24
Residuals	10	785		

Significant p-values (≤0.05, ≤0.01, ≤0.001) are indicated by one, two, or three asterisks, respectively. Sex = Medusa sex; Bp = Body part; df = degree of freedom; MS = mean of square; F = statistic F test.

Monthly prey selection calculated by Pearre’s selection index for all prey of *P*. *noctiluca* showed significant positive selection for copepods (November, May, and June), pteropods (November) and fish eggs (November, December, May, and June), and negative selection for ostracods (December) and furcilia (April) (Table 1). For other all prey types and months, *P*. *noctiluca* showed a non-selective foraging strategy.

### Stable Isotopes

**Figure 1:**
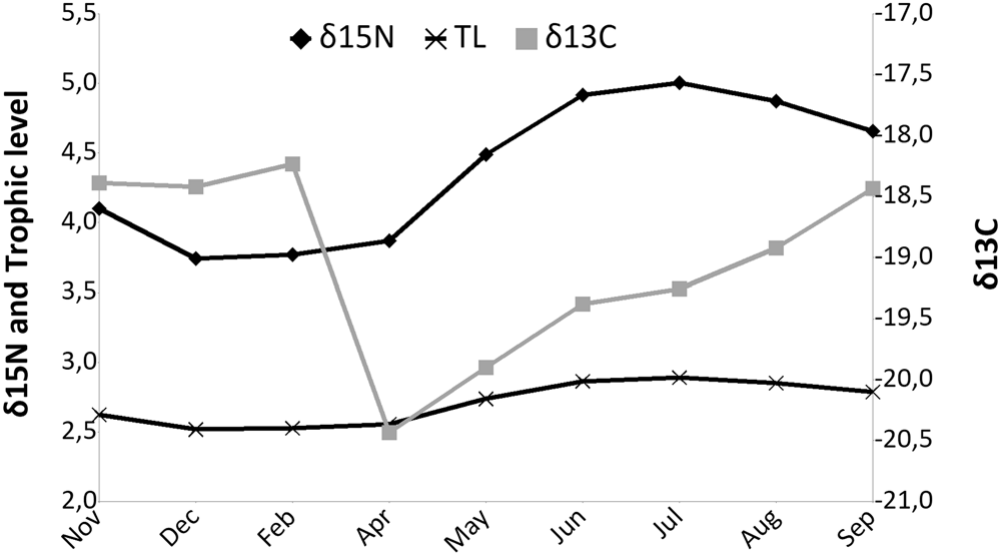
Monthly variations (mean ± standard error) of δ^13^C and δ^15^N (‰) values and trophic level (TL) in *Pelagia noctiluca* tissues.

Both δ^13^C and δ^15^N of *P*. *noctiluca* differed significantly over the year (Table 2). δ^13^C ranged from −20.5 ± 0.1‰ in April to −18.2 ± 0.1‰ in February (Fig. 1). In general, autumn and winter isotopic signatures had higher values than those in spring months (Fig. 1). δ^15^N ranged from 3.7 ± 0.1‰ in December to 5.0 ± 0.2‰ in July, with lower values in autumn and winter months (i.e., December and February) than in spring and summer (June, July, and August; Fig. 1).

Analysis of trophic level (TL) values (Fig. 1) showed the diet of *P*. *noctiluca* ranged from carnivorous in late spring-summer, with the highest TL value in July (2.8 ± 0.05), to omnivorous in late autumn-winter, with the lowest TL value in December (2.5 ± 0.02).

**Figure 2:**
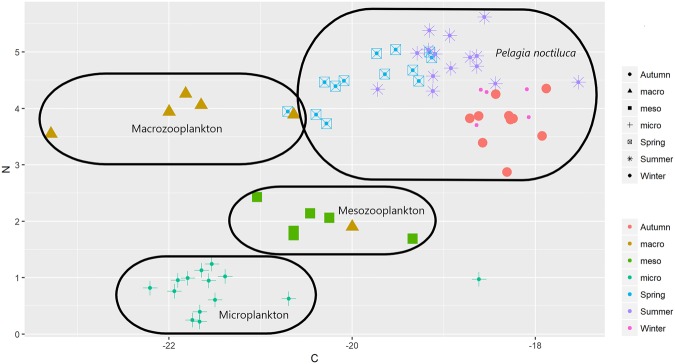
Bi-plot of stable isotope composition for *Pelagia noctiluca* in different season and size class of plankton preys.

Comparison of δ^15^N and δ^13^C isotopic compositions showed that *P*. *noctiluca* jellyfish and macrozooplankton (≥1000 µm) had similar δ^15^N values, highlighting a shared, high trophic level for these two categories (Fig. 2). ^15^N-depleted values of mesozooplankton (200–1000 µm) and microplankton (<200 µm) revealed a lower trophic level than jellyfish and macrozooplankton. Medusa samples from different periods of the year showed clear separation (Fig. 2) in three main clusters represented by jellyfish sampled in spring (April-May), spring-summer (June-July-August-September), or in autumn and winter months (November-December-February).

**Figure 3:**
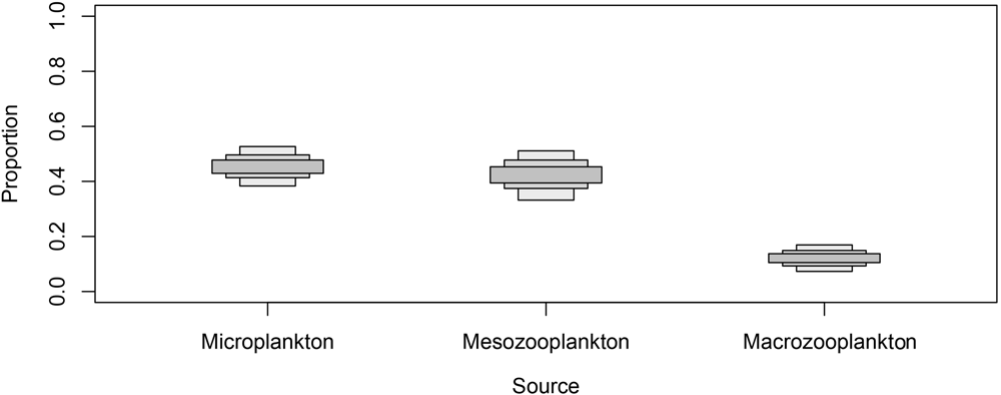
Modeled proportion of prey in the diet of *Pelagia noctiluca* medusae obtained using a stable isotope mixing model.

The results of the SIAR mixing model showed that *P*. *noctiluca* primarily consumed microplankton (95% credibility interval: 38–53%) and mesozooplankton (95% credibility interval: 33–51%) and, to a lesser extent, macrozooplankton (95% credibility interval: 7–17%) (Fig. 3).

### Fatty acids (FAs)

**Figure 4:**
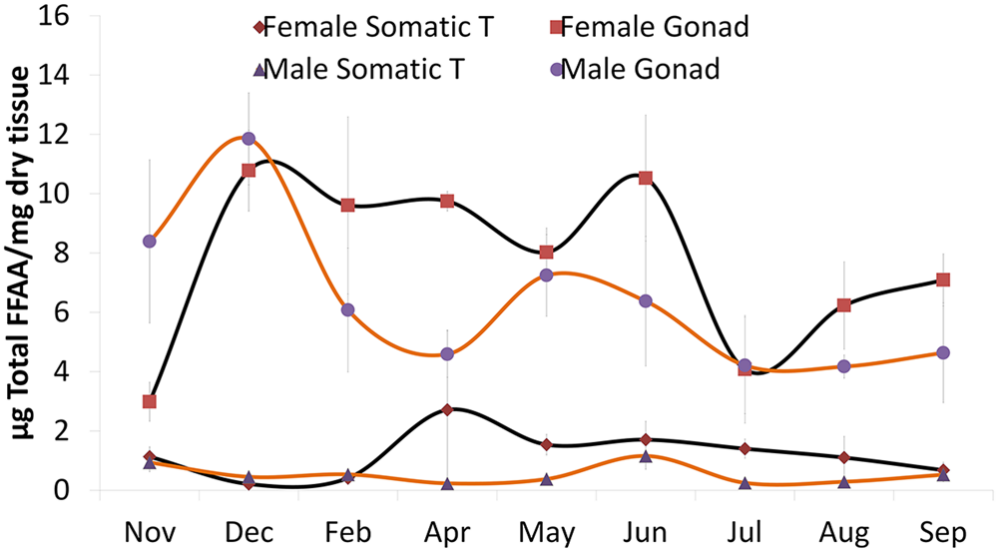
Monthly total fatty acid concentration (mean ± standard error) in different *Pelagia noctiluca* medusa tissues (somatic, gonad) and sex (male, female) from the Strait of Messina.

The FA concentration (µg of total FAs per mg of dry tissue) was higher and more variable in the gonads than in somatic tissue for both male and female jellyfish (Fig. 4). Female gonads accumulated FAs during three periods: spring (April: 10 ± 0.3, May: 8 ± 0.8 and June: 11 ± 2 µg fatty acid *mg^−1^ dry tissue), late summer (September: 7 ± 0.9 µg fatty acid * mg^−1^ dry tissue) and late autumn (December: 11 ± 1 µg fatty acid * mg^−1^ dry tissue), while male gonads had only two peaks (May: 7 ± 1 and December: 12 ± 2 µg fatty acid * mg^−1^ dry tissue) (Fig. 4).

The FAs encountered in the present work are presented in Supplementary Information 2. In general, polyunsaturated fatty acids (PUFAs) were the most abundant compounds, representing from 49.5% (November) to 68% (September) of total FAs. Major PUFAs were C20:4n-6 (Arachidonic acid; AA), C22:6n-3 (Docosahexaenoic acid; DHA), and C20:5n-3 (Eicosapentaenoic acid; EPA), which accounted for 37.5 to 41.6% of total FAs. Monounsaturated fatty acids (MUFAs) accounted for 2.3 to 26.3% of total FAs and consisted mostly of C16:1n-7 and C18:1(n-9). Saturated fatty acids (SFAs) were the next most abundant group, consisting mainly of C16:0, C17:0, and C18:0, which accounted for 24.8 to 28.1% of total FAs.

A 3-way PERMANOVA analysis on Log +1 transformed FA data suggested significant dietary shifts of *P*. *noctiluca* over the year (Table 2) and that FA composition of jellyfish differed significantly for the three factors considered: Month, Sex, and Body Part (Table 2).

SIMPER analysis showed that the FAs C16:0, C20:5n-6, C20:4n-3, and C22:6n-3 contributed most to the Bray-Curtis dissimilarity between Body Parts, between Sex and, in most cases, among months.

FA composition in zooplankton differed significantly among months (Table 2), although it did not differ among the zooplankton fractions. SIMPER analysis, conducted on a Bray Curtis similarity matrix, indicated homogeneity in FA composition among different spring zooplankton size classes (Similarity: 74.6%). C22:6n-3 contributed most to differentiate spring samples from other seasons, representing 29.42% of total FAs.

**Figure 5:**
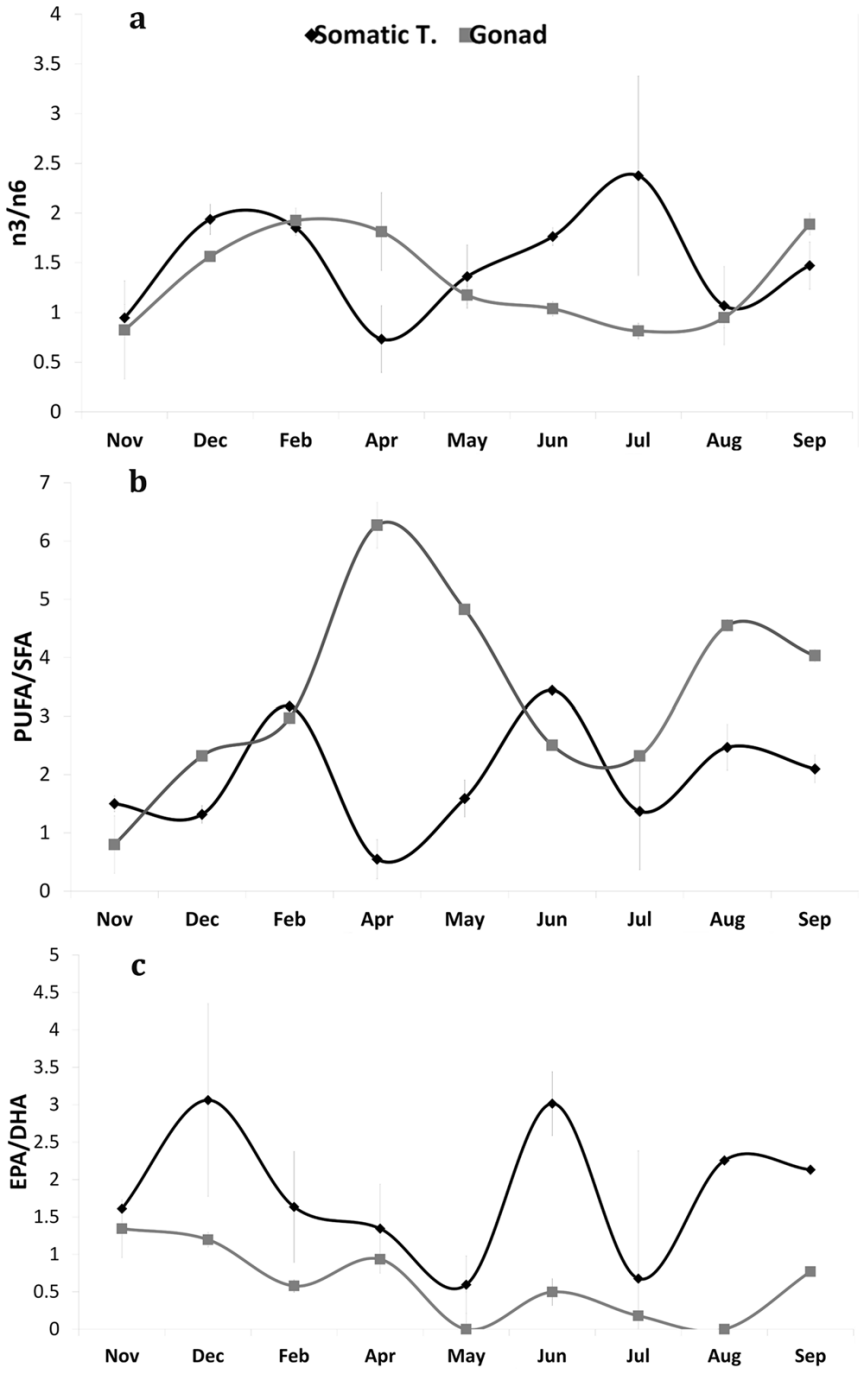
Monthly fatty acid variation (mean ± standard error) of (**a**) Omega 3/Omega 6 (n3/n6) ratio; (**b**) polyunsaturated fatty acid/saturated fatty acid (PUFA/SFA) ratio and (**c**) eicosapentaenoic acid/ docosahexaenoic acid (EPA/DHA) ratio in gonads and somatic tissue of *Pelagia noctiluca* medusae from the Strait of Messina.

The highest ratios of n-3/n-6 (Omega 3/Omega 6 FAs) were recorded in somatic tissue in July, followed by December and February and in the gonads in February-April, September, and December (Fig. 5a). PUFA/SFA (Polyunsaturated/Saturated fatty acid) were higher in June, August, and February in the somatic tissue and in April and August-September in gonads (Fig. 5b). The lowest EPA/DHA (Eicosapentaenoic/Docosahexaenoic acid) ratios were found in May and July in somatic tissues and in May and August in gonads (Fig. 5c).

## Discussion

The most available prey in the Strait of Messina, e.g., copepods, pteropods, fish eggs, and chaetognaths (Supplementary Information 1), were the main food sources for *Pelagia noctiluca* medusae in the present study. This jellyfish species has been described as a non-selective predator^27,29,36,37^, feeding on almost all types of zooplankton and ichthyoplankton^27,30,31,38^. The stomach contents of *P*. *noctiluca* showed a great variety of ingested prey, such as cladocerans, appendicularians, copepods, hydromedusae, siphonophores, and fish eggs. This result is in agreement with most of the above mentioned previous findings^27,29,39^.

Pearre’s selection index and data from the NW Mediterranean Sea^27^ concur that *P*. *noctiluca* in the Strait of Messina can be considered an unselective predator for most zooplankton taxa, capturing prey in proportion to their availability (Table 1). Apparently, euphausiid furcilia larvae and ostracods were negatively selected by *P*. *noctiluca*. However, this result may be biased by the diel vertical migration of those taxa, which are more commonly recorded in shallow water at night^40,41^, whereas during daytime when medusae were sampled, euphausiid larvae and ostracods move into deeper waters, possibly escaping from predation by jellyfish we collected. Also, the neutral selection of fish larvae as prey for *P*. *noctiluca* can be explained by an active anti-predator behavior, which can allow perception of slow predators and their avoidance^42,43^.

By contrast, fish eggs and copepods were positively selected as preferred prey, confirming the potential impact of *P*. *noctiluca* on fish recruitment either directly, by predation on fish eggs^44,45^, or indirectly by competition with fish larvae for the same food source (copepods)^46^. Such positive selection was most evident during the periods of sexual reproduction of *P*. *noctiluca*^35^, suggesting a correlation between high quality food and spawning periods. The high occurrence of fish eggs in the stomach of *P*. *noctiluca* might reflect the long digestion time for this prey type, as already known for *P*. *noctiluca* ephyrae^47^. Overall, these data corroborate the lack of strong prey selectivity in *P*. *noctiluca* medusae. Generalist predation may provide the simplest mechanistic explanation for the significant differences in *P*. *noctiluca* prey composition over the year, reflecting the seasonal availability of different prey and a clear opportunistic behavior.

δ^13^C and δ^15^N values of *P*. *noctiluca* jellyfish were compared with those of three different plankton size classes. Marine animals are 0–2‰ enriched in carbon isotopes^31,48^ and 3.2‰ enriched in nitrogen isotopes^49^ relative to those in their diets through the food web. Average δ^13^C values for jellyfish were about 1.8‰ higher than the values in mesozooplankton and fish eggs (200–1000 µm) and 2.4‰ higher than microplankton (<200 μm size class). Average δ^15^N values for jellyfishes were about 2.8‰ higher than the values in zooplankton 200–1000 μm size class and 3.6‰ higher than in microplankton <200 μm size class, indicating the enrichment of the heavy isotopes in the *P*. *noctiluca* tissues. Trophic fractionation of carbon isotope ratios between predators and their prey was considered to be ~2‰ for *P*. *noctiluca*^31^; thus, our results indicate that the main diet of *P*. *noctiluca* is composed of a mix of zooplankton size classes, which is consistent with the stomach content results. The same results come from our analysis of the nitrogen SI in *P*. *noctiluca*, with a rate of fractionation of 3.2‰. The isotopic fingerprint of microplankton detected in the medusa tissues can be the result of direct ingestion of bio-seston or indirect assimilation from the stomachs of mesozooplankton grazers that were consumed by *P*. *noctiluca*, like herbivorous copepods or appendicularians^50^. Grazing on microplankton is known for many cnidarians, such as *Aurelia* moon jellyfish from the eastern Atlantic, which ingested various microplankton taxa (e.g., particularly non-loricate ciliates, rotifers, flagellates, and others)^51^. Moreover, microplankton may represent an important food source for this jellyfish, particularly when mesozooplankton taxa are at low concentration^23,52^.

Analysis of seasonal variability of food preferences is uncommon for jellyfish. Our results suggest that δ^13^C in jellyfish varied from a high in autumn-winter months (~−18‰) to its lowest value in April (~−20‰). This variation may be related to the Strait of Messina having an alternating tidal regime driven by strong upwelling currents, with a seasonal peak from late spring to late summer^53^. Upwelled waters are characterized by a high micro-phytoplankton fraction^54,55^, with decreased δ^13^C and δ^15^N values at the base of the food web^56^. The seasonal upwelling peak may be followed by an opportunistic increase of direct consumption/indirect assimilation of micro-phytoplankton by *P*. *noctiluca* that is mirrored by the seasonal reduction of δ^13^C values in the jellyfish tissues. The SI results also agree with jellyfish stomach content data, in which most of the identifiable prey consisted of copepods and other herbivorous crustaceans. This can be interpreted as additional evidence of the lack of selection in the diet of *P*. *noctiluca* and the plasticity of its foraging and assimilation ability, because detritus is more abundant in autumn-early winter and diatom blooms are more abundant in late winter-spring months^55^.

High δ^15^N values and high trophic levels were found in summer in accordance with the increase in mesozooplankton abundance. Other studies on temporal changes in SI composition of dietary sources in food webs agree with the seasonal variation in C and N values in *P*. *noctiluca* tissues from the upwelling system in the Strait of Messina. Fukuda and Naganuma^57^ found that the diet of *Aurelia* medusae from the Sea of Japan shifted seasonally from a diatom-based food chain in spring to a detritus-based food chain in autumn. Recently, Javidpour *et al*.^33^ also suggested a temporal dietary shift from mesozooplankton to microplankton and re-suspended organic matter from the benthos for *A*. *aurita* from the Kiel Fjord (Baltic Sea). Other studies demonstrate feeding on microplankton^58–60^, but so far, only one study^59^ has demonstrated the ability of cnidarians to assimilate phytoplankton. Fresh material from algal blooms or reworked seston from resuspension or low quality material have different signals and are key to understand the trophic guilds of aquatic species^61^. *Pelagia noctiluca* can assimilate all zooplankton according to their abundance in the environment and smaller foods being digested in the stomach of its prey, utilizing an omnivorous assimilation strategy most of the year and an opportunistic carnivorous assimilation strategy during summer months.

The fatty acids also yielded results in accordance with all of the previous findings. Trophic biomarkers may be useful to understand the relationships of organisms in the food web and also to trace seasonal and ontogenetic changes in their physiological states^14,17^. Most of the previous research on lipid storage and FA metabolism in marine organisms dealt with taxa other than cnidarian jellyfish^62,63^. In addition, most studies on jellyfish FAs analyzed extracts from whole specimens without considering possible differences in FAs in different body parts, as appropriate to their respective physiological roles^64^. The study of trophic interactions through compositional analysis of fatty acids in different jellyfish tissues was carried out only for the colonial siphonophore *Nanomia cara*^16^, separated into two different body parts: the nectosome (specialized for swimming) and the siphosome (specialized for feeding and reproduction), with the siphosome yielding more fatty acids than the nectosome. Here we show for the first time the relationship between FAs and the diet of *P*. *noctiluca* medusae over a year, as well as the importance of considering FA extracts from somatic and gonadal tissues separately. The FA extracts from *P*. *noctiluca* soma and gonads highlighted their differences in terms of FA composition and storage capacity, respectively. The somatic tissue had low concentrations of FAs throughout the year, while the gonadal tissue had total FA concentrations that at times during the annual cycle exceeded ten times that in the somatic tissue of medusae, in agreement with previous findings based on other methods, e.g. on total biochemical composition, ash-free dry mass, or C:N ratios^65–67^.

Interestingly, EPA and AA values in the zooplankton fractions (9 ± 2 µg*mg^−1^dry tissue and 2 ± 3 µg*mg^−1^dry tissue, respectively) were lower than in *P*. *noctiluca* medusae (17 ± 2 µg*mg^−1^ dry tissue and 11 ± 1 µg*mg^−1^ dry tissue, respectively). This may be due to selective FA accumulation by jellyfish through indirect assimilation or direct predation of primary producers or microzooplankton (as shown by the gut contents). A comparable accumulation mechanism is known in other groups (e.g., copepods and cladocerans)^10,63^; however, molecular studies are now providing progress towards understanding the mechanisms of long-chain PUFA biosynthesis in marine invertebrates. Candidate genes for LC-PUFA biosynthetic enzymes (e.g., desaturases and elongases) have been confirmed from crustaceans and molluscs^63^. Thus, the possibility that part of the EPA and AA was generated through biosynthetic pathways in the medusa tissues, rather than representing assimilation from prey, needs to be investigated.

Palmitic Acid and DHA values were high in zooplankton samples, therefore jellyfish can accumulate these two FAs from the direct feeding on meso- and macrozooplankton^14,57^. The transfer of FAs from prey to *P*. *noctiluca* was not surprising. Siphonophores are known to assimilate over 90% of the carbon and nitrogen from their prey^68^. Therefore, the ability of selected FA assimilation and storage by *P*. *noctiluca* may be a crucial reproductive strategy, taking into consideration that part of FA energy will be used for the gonadal investment and the development of future ephyra stages.

FA biomarkers may provide complementary information about seasonal alternation between carnivory and omnivory. Their composition in *P*. *noctiluca* tissues varied for all factors considered in this study: SIMPER analysis highlighted the high contributions of C16:0 (Palmitic Acid), C20:4n-3 (Arachidonic Acid, AA), C20:5n-6 (Eicosapentanoic Acid, EPA), and C22:6n-3 (Docosahexaenoic Acid, DHA) in Bray-Curtis Dissimilarity between sex, body parts, and among months. EPA and DHA were most abundant in the gonads. These are essential FAs in fish broodstock diets, having critical functions as the main components of phospholipids of cell membranes^69^. The EPA/DHA ratio potentially may be used to determine the degree of carnivory, because DHA is highly conserved through the food web, being preferentially incorporated into polar lipids^70^. Consequently, the EPA/DHA ratio should decrease towards higher trophic levels^14^. Further, Cripps and Atkinson^71^ proposed that the ratio of polyunsaturated to saturated fatty acids (PUFA/SFA) in krill may be a useful indicator for carnivorous (high PUFA/SFA) versus herbivorous (low PUFA/SFA) feeding. In the gonads of *P*. *noctiluca*, both EPA/DHA and PUFA/SFA ratios suggest medium to high trophic levels (i.e., from secondary to tertiary consumer levels) for *P*. *noctiluca* medusae, in agreement with the results obtained from the SI analysis. Overall, the complementary information presented here reinforces the idea of a medium to high trophic position of *P*. *noctiluca* medusa, depending on the available food sources in the water column.

The reproductive tissues showed a wider seasonal oscillation of total FAs than did the somatic ones, related to the seasonal, biphasic cycle of spawning events observed in *P*. *noctiluca*^35^. The great accumulation capacity of FAs in the gonads may provide long-term signals of the quality and quantity of the diet, but the biochemical composition of gonads may be largely variable according to the reproductive cycle and the available food for an opportunistic species. Seasonal FA biomarker changes in jellyfish has been correlated with the population fitness throughout the year^17^. A strong seasonal pattern of FA biomarkers has been found in *Aurelia* moon jellyfish^57^, with lower quality FA composition in autumn (due to a prominent detritus-based nutrition) than in spring (due to a paramount diatom-based food source). This biochemical difference might be critical for the activation of sexual reproduction and eventually for increased larval supply and recruitment. In *Aurelia* and other scyphozoan with a benthic polyp stage, any increment of polyp populations will facilitate jellyfish outbursts by means of temperature-dependent strobilation timers^72^. Data on FAs stored in *P*. *noctiluca* medusae from the Strait of Messina demonstrated that energy flux was higher in spring-early summer and linked to the phytoplankton blooms, while the autumn decrease of n-3 FAs and increased abundance in SFAs (November-December, coincident with the refractory organic matter period^10^) was consistent with a prevalent detritus-based diet. The spring-early summer peak of copepods in the environment^54^ and in the stomach contents was also reflected in the amount of copepod-specific FA biomarkers. The abundance of copepods and fish eggs in the stomach contents is also reflected in the total FA concentration in the gonads, with a high energy investment for sexual reproduction.

In conclusion, stomach contents and biomarker analysis showed that *P*. *noctiluca* in the study area is not a selective predator, cyclically shifting its foraging strategy between carnivory and omnivory, depending on the seasonal availability of food items. Such cyclical changes seem to be tuned with the phenology of sexual reproduction of jellyfish, and to their abundance-size spectra. In turn, the high abundance of *P*. *noctiluca* may exert a top-down control on the zooplankton populations and the pelagic food webs.

Stomach contents and the selectivity index do not support for *P*. *noctiluca* in the Strait of Messina a role as main predator of fish larvae, as elsewhere hypothesized^30,37^. However, an impact on fish populations cannot be ruled out, as it may take place through three alternative, non-mutually exclusive ways: (1) direct top-down interaction by predation on fish eggs; (2) indirect competitive interaction with fish larvae, by dietary overlap for mesozooplankton as a food source; (3) indirect, bottom-up control, by grazing on microplankton so decreasing the available energy for upper trophic levels. The combination of SI and, potentially, FA analyses highlighted for the first time the importance of microzooplankton as prey for *P*. *noctiluca*, a neglected or underestimated role by direct stomach content analysis, showing that multiple mechanisms may coincide during jellyfish outbreaks to reduce food availability to higher trophic levels. The recognition of the opportunistic feeding behavior in a dominant, outbreak-forming species, such as *P*. *noctiluca*, corroborates the hypothesis that the variability of jellyfish trophic roles in plankton communities may be key both to their short-term ecological dominance and long-term evolutionary success^73^.

## Methods

### Ethics statement

No specific permits were required for the described field studies in the Strait of Messina. The species collected is the most common native jellyfish in the Mediterranean Sea and is not protected throughout its range. Sampling points did not include any protected or private lands.

### Study Area

The study was carried out in the Strait of Messina (Sicily, Italy). This site is strongly influenced by the peculiar hydrodynamic regime of the Strait, characterized by a six-hour alternation of northward (from the Ionian to Tyrrhenian seas) and southward (from the Tyrrhenian to Ionian seas) tidal currents, with upwelling and downwelling water masses reaching up to 200 cm s^−1^ speed^29^. The hydrodynamic complexity of the Strait ecosystem has a major influence on the spatial and vertical distribution of the organisms, especially on zooplankton communities^53^. The regular alternation of water masses prevents stratification of the water column and the formation of a summer thermocline. For this reason, the Strait is characterized by a higher productivity than other Mediterranean coastal areas.

In this study, surface water temperature was measured monthly using a bucket thermometer, and our data were implemented with daily time-series sea surface temperature downloaded from www.mareografico.it (ISPRA).

### Sampling

Live *P*. *noctiluca* specimens were collected two times per month from November 2011 to September 2012 with a 1-cm mesh hand net dipped from the boat. Due to adverse weather conditions and the absence of jellyfish from surface waters, we were unable to collect *P*. *noctiluca* in October 2011, January and March 2012. On board, jellyfish diameters were measured using a rule to the nearest mm. The specimens for stomach content analysis were fixed immediately after capture in 4% formaldehyde/water solution and stored individually in a container, to search all possible prey also in the formaldehyde/water solution, due to an event of stomach egestion during preservation.

Specimens for biomarker analysis were placed in a 50-l tank, with a continuous flow of seawater. Immediately after sampling (~2 hours), jellyfish were brought to the laboratory to proceed with morphometric and morphological analysis and sex identification^74^.

Three male and three female living jellyfish were selected from each sampling (i.e., six males and six females each month). The gonadal tissue was dissected from the somatic tissue for each specimen and each separately frozen in liquid nitrogen for subsequent trophic biomarker analyses. To obtain particulate organic matter for SI analysis, 3 liters of 200-µm-filtered sea water were re-filtered under vacuum through pre-ashed (4 h at 500 °C) Whatman GF/F glass fiber filters.

To provide additional information on *P*. *noctiluca* food availability and dietary preferences, two zooplankton samples, one each for taxonomic and biochemical analyses, were taken monthly in the same zone where jellyfish were sampled, using a WP2 net (40-cm mouth opening; 200 µm mesh size) with 10-minute surface horizontal hauls (1–2 m depth) at an average speed of 1 knot, in the same time and space of medusa samples. Zooplankton samples for taxonomic identification were fixed in 4% formaldehyde/water solution. Zooplankton samples for biochemical analyses were divided according into three size classes: <200 µm, 200–1000 µm and >1000 µm, and each fraction was filtered in pre-weighted GF-A filter.

### Stomach contents and stable isotope analysis

The recognizable prey taxa within the gastric pouches, manubrium, oral arms and mucus of 149 specimens of homogeneous size (see Table 1, including the number of samples per month) were identified to the finest possible taxonomic level with magnification of a stereomicroscope. Values of Pearre’s electivity index (*C*)^75^ were calculated for the most common zooplankton taxa by the numbers of prey m^−3^ and the average numbers of prey medusa^−1^ for each sample, using an ordinary chi-square test to assess their statistical significance.

Jellyfish somatic tissue (5 medusae for each month), zooplankton samples and filters with particulate organic matter were oven-dried at 60 °C and homogenized using mortar and pestle. To remove carbonate structures, one sub-sample of each sample for carbon isotope analysis in zooplankton and particulate organic matter was acidified by adding 1 M HCl drop-by-drop^76^. The samples were re-dried at 60 °C for 24 h.

All samples (medusa, zooplankton, and microplankton) were weighed in tin capsules, combusted and the resultant gases were analyzed in a Thermo Electron Isotope Ratio Mass Spectrometer for SI abundance. Internal laboratory standards were used after every five samples to calibrate the system. Isotopic values were expressed in δ notation as parts per thousand (‰) differences from international standards (Vienna Pee Dee Belemnite and atmospheric N2 for carbon and nitrogen respectively):

1 $$\delta X=[(\frac{{R}_{sample}}{{R}_{standard}})-1]\ast {10}^{3}$$

where *X* is equal to ^15^N or ^13^C and *R* is the corresponding ratio ^13^C/^12^C or ^15^N/^14^N. Based on replicates of laboratory standards, analytical precision was ±0.2 and ± 0.1‰ for δ^15^N and δ^13^C respectively. Trophic levels (TL) of *P*. *noctiluca* were estimated assuming an average increase in δ^15^N values of 3.2‰ between successive trophic levels^49,77^, and an average increase in δ^13^C values of 2‰ between successive trophic levels^31^. In agreement with^23^, we did not use the trophic enrichment factors value for nitrogen proposed by D’ Ambra *et al*.^78^, because the trophic level of *P*. *noctiluca* would result in unrealistically high trophic positions.

TL were estimated according to the following formula:

2 $$TF=[\frac{{\delta }^{15}{N}_{f}-{\delta }^{15}{N}_{ref}}{3.2}]+T{F}_{ref}$$

where δ^15^N_f_ and δ^15^N_ref_ are the nitrogen isotopic signature of the medusa and a baseline reference organism respectively; 3.2 is the δ^15^N expected isotopic fractionation per trophic level; TF_ref_ is the trophic level of the baseline organism. In this study the baseline organisms were the mesozooplanktonic calanoid copepods (TF_ref_ = 2).

### Fatty acid extraction

Approximately 100 mg dry weight (±0.01) of medusa tissue (Gonad or Somatic tissue) and 50 mg dry weight (±0.01) of different zooplankton size fractions were extracted by microwave-assisted extraction (5 min at 70 °C) with 10 mL of 3:1 dichloromethane-methanol, and using 2-octyldodecanoic acid and 5β-cholanic acid as internal standards. The extract was taken to near dryness in a centrifugal vacuum concentrator at a constant temperature and fractionated by solid phase extraction according to Ruiz *et al*.^79^. The sample was re-dissolved on 0.5 mL of chloroform and eluted through a 500 mg amino-propyl column previously conditioned with 4 mL of n-hexane. The first fraction was eluted in 3 mL of chloroform: 2-propanol (2:1), and the fatty acids recovered in 8.5 mL of diethyl ether: acetic acid (98:2). The FA fraction was methylated using a solution of 20% methanol/BF3 heated at 90 °C for 1 h. The reaction was quenched with 4 mL of water saturated with NaCl. The methyl esters of FFA were recovered by extracting twice in 3 mL of n-hexane. The combined extracts were taken to near dryness, re-dissolved in 1.5 mL of chloroform, eluted through a glass column filled with Na_2_SO_4_ to remove residual water, taken to dryness under a gentle nitrogen flux, and stored at −20 °C until analysis. The samples were re-dissolved in 40 μl (for medusa tissue) or 100 μl (for zooplankton size fraction) of isooctane. Gas chromatographic analysis was performed with a Thermo Finnigan Trace Gas Chromatography Ultra instrument equipped with a flame ionization detector, a splitless injector and a DB–5 Agilent column (30 m length, 0.25 mm internal diameter and 0.25 μm phase thickness). Helium was used as a carrier gas at 33 cm s^−1^. The oven temperature was programmed to increase from 50 °C to 320 °C at 10 °C^−min^. The injector and detector temperatures were 300 °C and 320 °C, respectively. The methyl esters of fatty acids were identified by comparing their retention times with those of FA standards (37 FAME compounds, Supelco^®^ Mix C4-C24). Fatty acids were quantified by integrating areas under the peaks in the gas chromatograph traces (Chromquest 4.1 software) with calibrations derived from internal standards^80^. The Mix C4-C24 satisfied almost all the fatty acid identification; only in few cases GC-MS has been used to identify unknown peaks.

### Statistical analysis

Differences in the total numbers of prey in the stomach contents of each jellyfish (number of prey medusae^−1^) among months were analyzed by one-way analysis of variance (ANOVA), after testing homogeneity of variance by Cochran’s test, while differences of monthly diet composition were analyzed by one-way Permutational Multivariate ANOVA (PERMANOVA^81^) performed on a triangular matrix based on Euclidean distances.

To define the diet of *P*. *noctiluca* based on δ^13^C and δ^15^N of medusae and their potential prey, we computed a mixing model using the package SIAR^82^ (version 4.1) downloaded with R (“R: A language and environment for statistical computing” 2013; version 2.13.1) from the Comprehensive Archive Network site (CRAN, http://cran.r-project.org/). The mixing model was based on prey composition in the predator’s diet inferred from all feasible prey. The proportional diet composition of medusae was estimated using the following fractionation values: δ^13^C 2 + 0.3‰^31^, and δ^15^N 3.2 + 0.1‰^49^. Moreover, differences in δ^13^C and δ^15^N values among different sampled months, were investigated separately using one-way ANOVA. The total amount of FAs was investigated using 3-way PERMANOVA. In this case the independent variables used were: Month (fixed with 8 levels), Sex (fixed and orthogonal with two levels), and Body part (fixed and orthogonal with two levels: gonadic and somatic tissue). Differences in FA composition of medusae among Months, Sex, and Body parts were tested using 3-way PERMANOVA based on Bray-Curtis similarities matrix of log +1 transformed data. In this case we preferred the use of Bray-Curtis similarities matrix because of the large number of variables considered (FAs) and the presence of zero, following the recommendation of Legendre & Legendre^83^. The SIMPER routine was used to identify the dissimilarity among months, as well as the FAs responsible for the observed differences. FA composition of zooplankton sampling were analyzed using 2-way PERMANOVA, in order to highlight differences among different months and zooplankton size and their interaction.

All analyses were performed using PRIMER6 & PERMANOVA+^84,85^ and R (version 2.13.1; CRAN, http://cran.r-project.org/).

## Electronic supplementary material

Supplementary Information
