# Supplementary material for: Seasonal variability of diet and trophic level of the gelatinous predator Pelagia noctiluca (Scyphozoa)
Source: Sci Rep. 2018 Aug 14;8:12140. doi: 10.1038/s41598-018-30474-x (PMC6092325; doi:10.1038/s41598-018-30474-x)
Supplement: Supplementary file 1 — Supplementary Information [file 41598_2018_30474_MOESM1_ESM.pdf]

Seasonal variability of diet and trophic level of the gelatinous predator *Pelagia noctiluca* (Scyphozoa)

Giacomo Milisenda [1,2*], Sergio Rossi[2,3], Salvatrice Vizzini[2,4], Veronica L. Fuentes[5], Jennifer E. Purcell[6], Uxue Tilves[5], Stefano Piraino[2,3*].

[1]Institute for Coastal Marine Environment (IAMC), National Research Council (CNR), Via L. Vaccara n 61, Mazara del Vallo (TP), 91026, Italy.

[2]CONISMA - Consorzio Nazionale Interuniversitario per le Scienze del Mare, 00196, Rome, Italy.

[3]Department of Biological and Environmental Sciences and Technologies, University of Salento, 73100, Lecce, Italy.

[4]Department of Earth and Marine Sciences, University of Palermo, via Archirafi 18, 90100, Palermo, Italy.

[5]Institut de Ciències del Mar, ICM-CSIC, PG. Maritim de la Barceloneta, 08003 Barcelona, Spain

[6]Western Washington University, Department of Biology, Bellingham, WA 98225, USA.

[*]Corresponding authors: giacomo.milisenda@gmail.com; stefano.piraino@unisalento.it

	Sep	Nov	dec	Feb	Apr	may	Jun	Jul
Copepods	435.0	247.3	1258.7	419.0	165.7	486.6	709.9	291.6
Cladocerans	10.5	1.2	0.0	0.0	0.0	46.5	56.7	90.6
Appendicularians	8.4	16.1	23.2	11.2	10.3	73.2	9.2	30.8
Chaetognaths	3.0	13.0	18.5	32.8	1.1	3.5	34.0	8.8
Ostracods	10.5	7.2	48.9	10.5	8.6	4.7	2.8	0.6
Siphonophores	2.4	7.6	5.4	15.8	1.6	7.3	11.5	14.9
Pteropods	1.3	2.5	20.3	3.5	0.4	0.0	3.9	11.4
Hydromedusae	0.4	1.9	2.1	6.0	0.9	3.0	0.8	1.8
Furcilia	1.1	0.8	0.9	3.9	0.2	0.7	0.6	1.2
Fish eggs	0.5	0.1	0.1	0.3	0.2	0.2	6.7	2.2
Fish larvae	0.9	0.0	0.0	0.3	0.1	0.2	0.6	1.0
Others	3.8	8.3	7.6	16.9	11.1	8.0	3.6	9.3

SM1. Prey abundance (number m^{-3})

	Somatic tissue				Gonadic tissue			
	female		male		female		male	
	mean	SE	mean	SE	mean	SE	mean	SE
Lauric Acid Methyl Ester (C 12:0)	0.14	0.09	0.22	0.10	0.07	0.05	0.09	0.07
Tridecanoic Acid Methyl Ester (C13:0)	0.14	0.08	0.22	0.09	0.04	0.03	0.08	0.05
Myristoleic Acid Methyl Ester (C14:1)	0.06	0.05	0.18	0.08	0.01	0.02	0.02	0.02
Myristic Acid Methyl Ester (C14:0)	0.00	0.00	0.00	0.00	0.00	0.00	0.00	0.00
cis-10-Pentadecanoic Acid Methyl Ester (C15:1)	0.01	0.01	0.04	0.02	0.00	0.01	0.00	0.00
Pentadecanoic Acid Methyl Ester (C15:0)	0.92	0.25	1.52	0.28	0.89	0.20	0.76	0.19
Palmitoleic Acid Methyl Ester (C16:1 n-7)	1.01	0.21	1.44	0.23	1.25	0.29	1.10	0.17
Palmitic Acid Methyl Ester (C16:0)	12.42	0.78	25.63	0.95	17.19	0.83	15.14	0.78
cis-10-Heptadecanoic Acid Methyl Ester (C17:1)	0.02	0.04	0.00	0.01	0.05	0.04	0.01	0.02
Heptadecanoic Acid Methyl Ester (C17:0)	2.00	0.37	3.24	0.40	2.70	0.23	2.18	0.35
γ-Linoleic Acid Methyl Ester (C18:3n6)	0.17	0.11	0.17	0.10	0.15	0.08	0.10	0.06
stearidonic acid (SDA) (C18:4n3)	2.43	0.35	2.10	0.27	2.86	0.33	2.18	0.21
Linolelaidic Acid Methyl Ester (C18:2 n6t)	1.65	0.25	2.20	0.24	1.66	0.20	1.65	0.14
Linolenic Acid Methyl Ester (C18:3n3)	0.59	0.18	0.38	0.16	0.72	0.19	0.54	0.09
Elaidic Acid Methyl Ester (C18:1n9t)	3.94	0.51	7.88	0.61	3.08	0.30	3.40	0.24
Linoleic Acid Methyl Ester (C18:2n6c)	0.24	0.14	1.38	0.23	0.24	0.14	0.14	0.06
Oleic Acid Methyl Ester (C18:1n9c)	3.15	0.43	4.38	0.57	2.33	0.28	2.54	0.30
Stearic Acid Methyl Ester (C18:0)	6.03	0.57	10.05	0.79	5.73	0.37	3.46	0.26
cis-5,8,11,14,17-Eicosapentanoic Acid Methyl Ester (20:5n3)	14.60	0.80	10.09	0.81	7.34	0.67	6.58	0.32
Arachidonic Acid Methyl Ester (C20:4n6)	18.50	0.85	8.95	0.73	21.19	0.69	25.25	0.36
cis-11,14,17-Eicosatrienoic Acid Methyl Ester (C20:3n3)	0.70	0.18	0.26	0.14	3.58	0.52	0.97	0.19
cis-11,14-Eicosadienoic Acid Methyl Ester (C20:2)	0.44	0.13	0.43	0.15	0.36	0.10	0.48	0.09
cis-11-Eicosenoic Acid Methyl Ester (C20:1)	0.39	0.13	0.66	0.18	0.28	0.10	0.37	0.12
Arachidic Acid Methyl Ester (C20:0)	0.24	0.11	0.49	0.17	0.22	0.06	0.11	0.06
Heneicosanoic Acid Methyl Ester (C21:0)	0.43	0.16	0.45	0.13	0.12	0.07	0.04	0.04
cis-4,7,10,13,16,19-Docosahexaenoic Acid Methyl Ester (C22:6n3)	9.72	0.80	5.55	0.67	13.16	0.73	17.13	0.76
docosatetraenoic acid (22: 4n− 6)	3.98	0.45	2.39	0.31	3.75	0.38	2.70	0.24
docosapentaenoic acid (DPA) (22:5, n-3)	5.12	0.43	2.68	0.48	4.82	0.42	6.64	0.31
cis-13,16-Docosadienoic Acid Methyl Ester (C22:2)	0.34	0.18	0.32	0.15	0.30	0.12	0.09	0.05
Erucic Acid Methyl Ester (C22:1n9)	0.60	0.19	1.37	0.25	0.17	0.06	0.18	0.10
Behenic Acid Methyl Ester (C22:0)	0.90	0.29	0.96	0.28	0.04	0.05	0.03	0.02
Tricosanoic Acid Methyl Ester (C23:0)	0.41	0.16	0.31	0.12	0.10	0.05	0.05	0.04
Tetracosatetraenoic acid (C24:4n-6)	2.37	0.47	1.11	0.26	1.10	0.19	1.00	0.12
Tetracosapentaenoic acid(C24:5n-3)	5.34	0.50	1.99	0.39	3.85	0.40	4.66	0.37
Nervonic Acid Methyl Ester (24:1)	0.43	0.18	0.66	0.21	0.53	0.16	0.30	0.09
Lignoceric Acid Methyl Ester (C24:0)	0.58	0.18	0.29	0.15	0.09	0.06	0.04	0.04

SM2. List of fatty acids encountered in different tissue (µg of total FAs per mg of dry tissue). SE= standard error.
